# Telephone and 'Straight-to-Test' Model in Triaging Urgent Suspected Cancer Referrals in a Head and Neck Clinic

**DOI:** 10.7759/cureus.114075

**Published:** 2026-08-06

**Authors:** Maria Kiakou, Billy LK Wong, Ioannis Chatzistefanou, Mark Puvanendran

**Affiliations:** 1 Ear, Nose and Throat (ENT), St George's Hospital, London, GBR; 2 Otolaryngology - Head and Neck Surgery, East Suffolk and North Essex NHS Foundation Trust, Ipswich, GBR; 3 Oral and Maxillofacial Surgery, Aristotle University of Thessaloniki (AUTH), Athens, GRC; 4 Otolaryngology, Mid and South Essex NHS Foundation Trust, Chelmsford, GBR

**Keywords:** head and neck cancer (hnc), radiological investigations, straight-to-test, telephone triage, urgent suspected cancer referrals

## Abstract

Background: In the face of challenging times characterized by high referral volumes, limited resources, and service constraints, our Head and Neck Cancer service at the Broomfield Hospital (Mid and South Essex NHS Foundation Trust) adopted a telephone triage system to streamline patient management and prioritise high-risk cases. To improve efficiency and expedite cancer pathways, we implemented a telephone triage and 'straight-to-test' model (STT), whereby high-risk patients are referred directly for diagnostic imaging following telephone triage, bypassing an initial face-to-face consultation. This study evaluated the effectiveness of this approach in a regional Head and Neck Cancer hub, with the malignancy detection rate as the primary outcome.

Methods: We conducted a retrospective observational pilot study at a regional Head and Neck Cancer hub in the United Kingdom. The study included 94 consecutive patients referred via the urgent two-week wait (2WW) pathway for suspected head and neck cancer. All patients underwent an initial telephone consultation with an Ear, Nose, and Throat (ENT) clinician, during which they were triaged and referred directly for appropriate imaging investigations, typically cross-sectional imaging, without a prior face-to-face clinical assessment. Patient demographics, imaging modality, final diagnosis, and clinical outcomes were collected. The primary outcome was the malignancy detection rate using this telephone triage and STT/telephone-and-test (TnT) model. Secondary outcomes included imaging utilisation, discharge following imaging, and the requirement for subsequent face-to-face assessment.

Results: The study included 40 (42.6%) male and 54 (57.4%) female patients, with a mean age of 56.2 ± 17.6 years (range, 19-89 years). During the study period, 495 two-week wait referrals were received. Of these, 104 (21.0%) patients were discharged following their initial telephone consultation. Ninety-four patients (19.0%) were referred directly for imaging investigations after telephone triage through the STT pathway. Malignancy was identified in 14 (14.9%) patients and included squamous cell carcinoma, well- differentiated thyroid carcinoma, lymphoproliferative disease, and metastatic malignancy. A further 40.4% (n=38) of patients were diagnosed with benign conditions, including benign salivary gland tumours, benign thyroid disease, and physiological lymphadenopathy. Following radiological investigation, 49 (52.1%) patients were discharged without requiring any further consultations. Cross-sectional imaging (CT/MRI) was performed significantly more frequently in patients with malignant pathology than in those with non-malignant findings (50.0% vs. 12.5%, p=0.003).

Conclusion: The telephone triage and straight-to-test approach is an effective model for managing head and neck 2WW referrals. It enables early identification of malignant disease while reducing unnecessary outpatient appointments and may be safely implemented in high-volume centres, particularly where clinical resources are limited.

## Introduction

The COVID-19 pandemic had a profound impact on healthcare services. Healthcare systems were required to simultaneously manage the pandemic while maintaining the delivery of essential medical care. Despite unprecedented pressure, workforce exhaustion, and significant operational challenges, the NHS successfully adapted to these demands and mitigated substantial risks [1]. When viewing each challenge as an opportunity for reflection and improvement, the COVID-19 era provided valuable lessons in crisis management, service resilience, and adaptability. It also accelerated the adoption of innovative approaches to managing increasing demand within healthcare services. Furthermore, analysis of data accumulated during the pandemic has generated important scientific evidence that can inform future service delivery and improve preparedness for similar public health challenges [1].

During the pandemic, NHS England published guidance recommending the use of telephone triage to minimise face-to-face interactions and reduce outpatient appointments across medical specialties [2]. Similarly, the British Association of Head and Neck Oncologists (BAHNO), in collaboration with ENT UK, issued joint guidance recommending the prioritisation of patients with a high suspicion of malignancy while safely deferring those considered to be at lower risk. Where feasible, a one-stop service model was encouraged [3].

Four years after the onset of the pandemic, Head and Neck Cancer services continue to experience considerable pressure owing to increasing demand for urgent cancer referrals through the two-week wait (2WW) pathway. Rising numbers of GP referrals, an ageing population, and increasing healthcare demands have all contributed to this sustained pressure [4]. In response, many hospitals have retained and expanded telephone triage clinics, delivered by either clinicians or Head and Neck specialist nurses, building on service models introduced during the COVID-19 pandemic. Patients are risk-stratified into low- and high-risk groups using the Head and Neck Cancer Risk Calculator (HaNC-RC), version 2, allowing prioritisation according to the estimated risk of malignancy [5,6].

Despite these developments, healthcare services continue to face additional challenges, including industrial action, workforce shortages, staff sickness, and financial constraints, prompting the exploration of alternative methods to improve the efficiency of cancer pathways. Building on the experience gained during the pandemic, we explored the feasibility of referring selected patients directly for diagnostic imaging following telephone triage, before any face-to-face clinical assessment, with the aim of expediting the cancer pathway. Similar straight-to-test (STT) pathways are already well established in several other medical specialties.

Before considering wider implementation of this pathway, we believed it was important to review and evaluate our COVID-era experience, during which this approach had already been adopted. Although the effectiveness of remote triage for suspected head and neck cancer using the HaNC-RC, version 2, has been well established and extensively reported in the literature [5], the effectiveness of combining telephone triage with immediate imaging has not been fully evaluated.

This study therefore assesses the feasibility and clinical utility of a pilot 'telephone-and-test' (TnT) pathway, in which telephone consultation is combined with direct imaging for patients referred through the urgent suspected head and neck cancer pathway. Using retrospectively collected data, we aim to trial the TnT pathway and assess its ability to identify clinically significant pathology, including malignancy, its impact on radiology utilisation, and its potential role as a safe triage strategy for contemporary head and neck cancer services.

## Materials and methods

This study was conducted at a regional Head and Neck Cancer hub (Mid and South Essex NHS Foundation Trust) serving a population of more than 1.2 million people. The retrospective observational cohort study was performed using prospectively maintained service data collected during the earliest and most restrictive phase of the COVID-19 pandemic, between April 2020 and November 2020. Consecutive patients who were referred under the urgent suspected cancer pathway (2WW), who were particularly triaged to the TnT pathway after consultant-led telephone triage, were included in the study. All telephone consultations were conducted by consultant otolaryngologists.

Patients were risk-stratified using the HaNC-RC (version 2) according to their presenting symptoms. The HaNC-RC scores were recorded for all patients. Patients with a predicted cancer risk of <2% were deemed as low-risk and were discharged to primary care, with appropriate safety-netting advice but without a scheduled follow-up, while patients requiring urgent face-to-face assessment were offered an expedited outpatient appointment. Carefully selected patients, including those presenting with a neck lump, dysphagia, odynophagia, B symptomatology, or previously abnormal investigations requested by primary care or other specialties, were referred directly and urgently (under the 2WW pathway) for the most appropriate imaging modality without an initial face-to-face consultation. The selection of the most appropriate imaging modality was decided by individual consultant judgement; however, it generally followed the standardised guidelines of our local multidisciplinary team (MDT). This means that patients with a neck lump had a US-guided biopsy +/- MRI neck, individuals with swallowing problems were investigated with a contrast swallow and CT of the neck and chest, and thyroid or parotid lesions were explored further with US of the thyroid or parotid. These patients were included in this straight-to-test/telephone-and-test cohort of the present study.

Patient demographics, including age and sex, imaging modality, imaging findings, final diagnosis, and clinical outcomes, were extracted from electronic medical records. Final diagnoses were categorised as no significant abnormality, benign pathology, or malignant pathology according to the radiological findings and histopathology or cytology, where applicable.

The primary outcome was the malignancy detection rate of the TnT pathway, while secondary outcomes included final diagnosis, imaging utilisation, discharge rate following imaging, and the requirement for subsequent face-to-face assessment.

Continuous variables are presented as means ± standard deviations (SDs) and categorical variables are presented as frequencies and percentages (n, %). The independent-samples t-test was used for comparisons between patients with malignant and non-malignant pathology for continuous variables and Fisher's exact test for categorical variables. A p-value of <0.05 was considered statistically significant. Statistical analyses were performed using IBM SPSS Statistics for Windows, version 29.0 (IBM Corp., Armonk, USA).

The study was reported in accordance with the Standards for Quality Improvement Reporting Excellence (SQUIRE) 2.0 guidelines [7].

## Results

**Figure 1:**
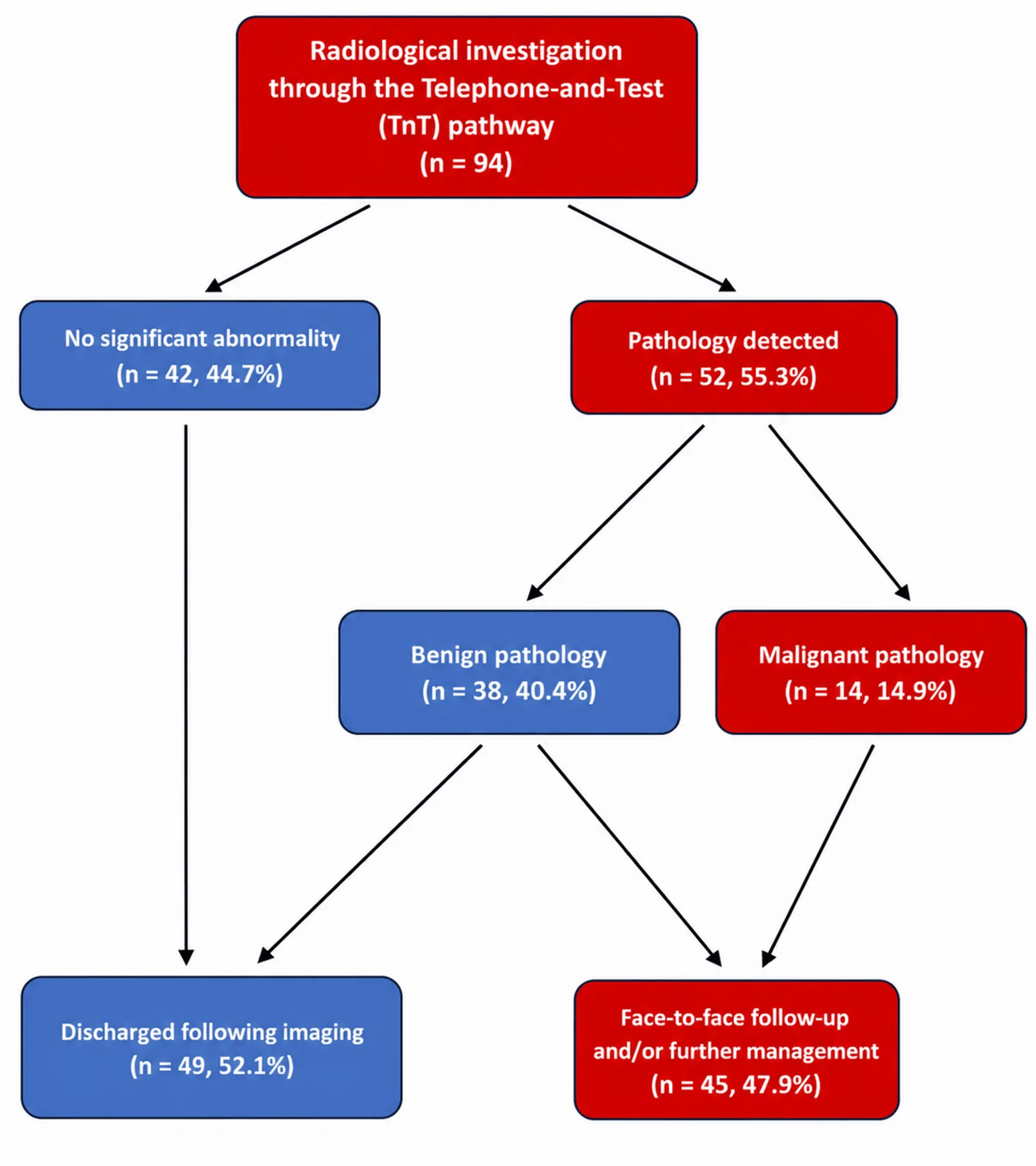
Radiological investigation pathway flowchart for the cohort

During the study period, we received 495 urgent suspected head and neck cancer referrals. Following telephone triage conducted by an ENT consultant, 104 (21.0%) patients were discharged with safety-netting advice, 127 (25.7%) were downgraded to routine outpatient review, and 180 (36.4%) were offered an urgent face-to-face consultation. A total of 94 (19.0%) patients were referred directly for radiological investigation through the TnT pathway without an initial face-to-face assessment (Figure 1).

The basic demographic characteristics, the different imaging modalities, final diagnoses, and clinical outcomes of patients managed through the TnT pathway are summarised in Table 1.

**Table 1:** Basic demographic characteristics, imaging modalities, final diagnoses, and clinical outcomes of patients managed through the telephone-and-test (TnT) pathway (n=94)

Characteristic	Value
Demographic characteristics	
Age, years, mean ± SD (range)	56.2 ± 17.6 (19-89)
Male	40 (42.6%)
Female	54 (57.4%)
Imaging investigations	
Neck ultrasound	44 (46.8%)
Thyroid ultrasound	16 (17.0%)
Parotid/submandibular gland ultrasound	7 (7.4%)
Computed tomography (CT)	15 (16.0%)
Magnetic resonance imaging (MRI)	4 (4.3%)
Barium swallow	11 (11.7%)
Ultrasound-guided biopsy	25 (26.6%)
Final diagnosis	
No significant abnormality detected	42 (44.7%)
Benign pathology	38 (40.4%)
Malignant pathology	14 (14.9%)
Clinical outcome	
Discharged following imaging	49 (52.1%)
Required further assessment/follow-up	45 (47.9%)

The percentages do not total 100%, as some patients underwent more than one imaging investigation. Final diagnosis was based on radiological findings together, where applicable, with subsequent histopathological/cytological confirmation.

In the studied cohort of patients, 40 (42.6%) were male and 54 (57.4%) were female patients, with a mean age of 56.2 ± 17.6 years (range, 19-89 years). The most frequently requested imaging investigation was the ultrasound of the neck, which was performed in 44 (46.8%) patients, followed by focused thyroid ultrasound in 16 (17.0%), neck (+/- chest) CT in 15 (16.0%), barium swallow in 11 (11.7%), focused salivary gland ultrasound in 7 (7.4%), magnetic resonance imaging (MRI) in 4 (4.3%), and combined imaging modalities in 4 (4.3%) patients. Ultrasound-guided biopsy was performed in 25 (26.6%) patients.

**Figure 2:**
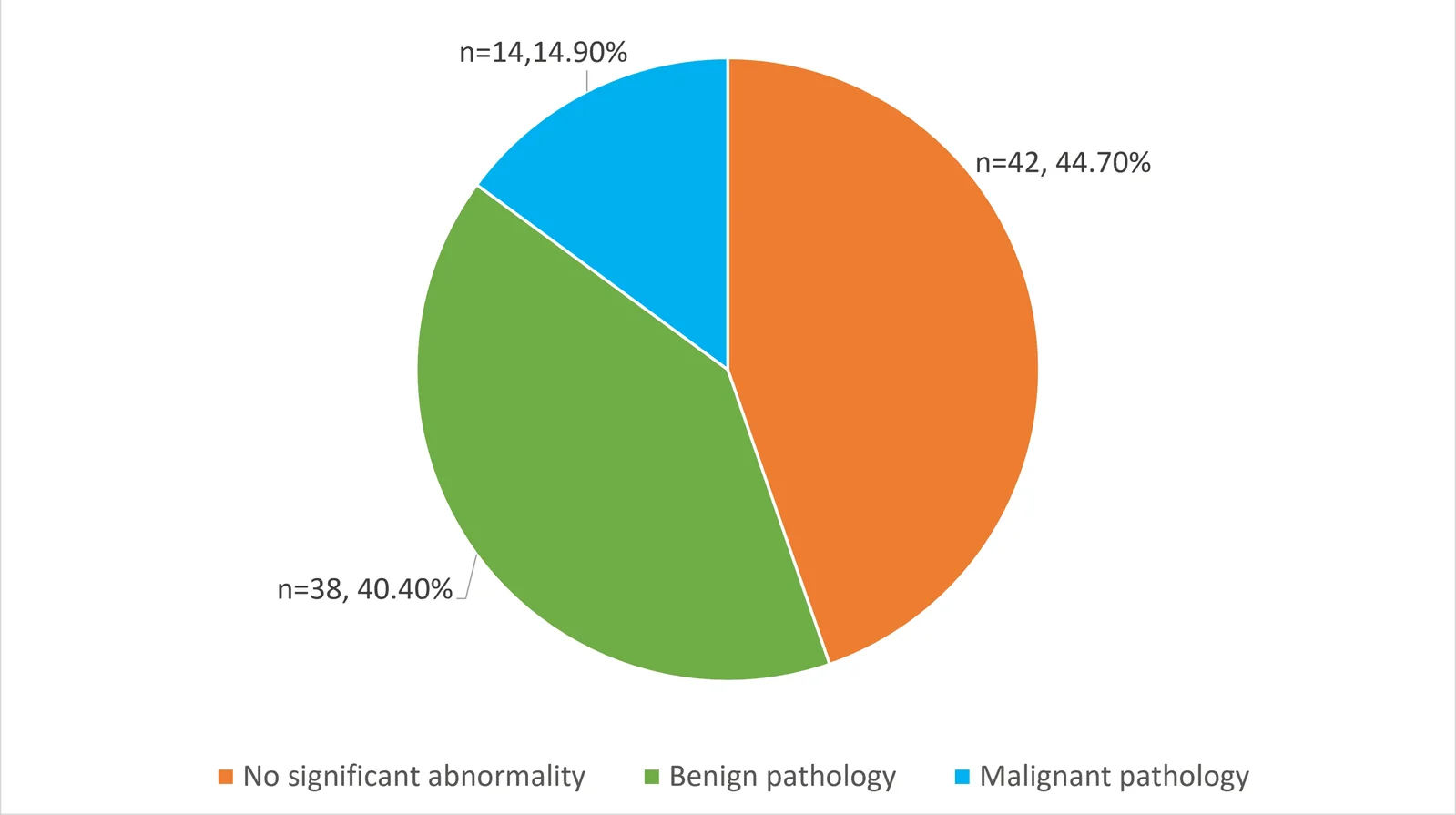
Distribution of final diagnoses

The distribution of final diagnoses is summarised in Table 1 and illustrated in Figure 2.

**Figure 3:**
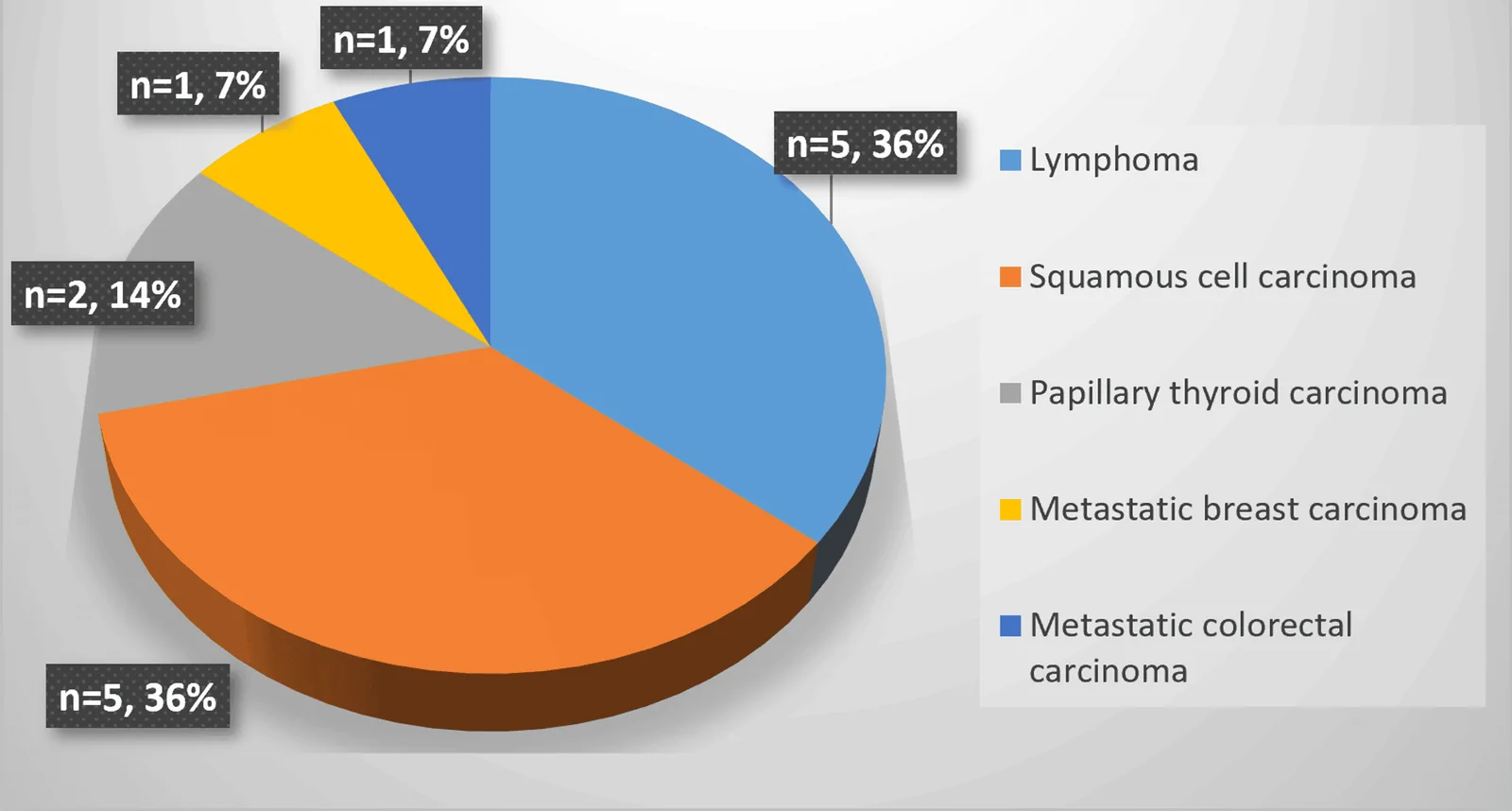
Distribution of malignant diagnoses

Following completion of radiological investigations, 49 (52.1%) patients were discharged without requiring further outpatient review, while 45 (47.9%) were streamlined to further face-to-face assessment. Beyond imaging, indications for further review included persistent or unresolved symptoms, need for examination with flexible naso-endoscopy, surgical management, or patient preference. A total of 14 (14.9%) patients from the straight-to-test cohort were identified to have malignant disease, but only half of them had a diagnosis of a cancer of the head and neck region. The rest of the diagnoses were metastatic disease from other non-head and neck primaries (n=2, 14%) or haematological malignancies (n=5, 36%) as shown in Figure 3.

A comparison of demographic and imaging characteristics between patients with malignant and non-malignant pathology is summarised in Table 2.

**Table 2:** Comparison of demographic and imaging characteristics between patients with malignant and non-malignant pathology

Variable	Malignant (n=14)	Non-malignant (n=80)	p-value
Age, years, mean ± SD	58.6 ± 17.2	55.8 ± 17.7	0.579
Male sex	7 (50.0%)	33 (41.3%)	0.570
Cross-sectional imaging (CT/MRI)	7 (50.0%)	10 (12.5%)	0.003

A comparison between patients with malignant and non-malignant pathology demonstrated no significant differences in age or sex. The mean age of patients diagnosed with malignant disease was 58.6 ± 17.2 years compared with 55.8 ± 17.7 years in those with non-malignant findings (p=0.579). Similarly, the proportion of male patients did not differ significantly between groups (7 (50.0%) vs. 33 (41.3%), p=0.570).

In contrast, cross-sectional imaging (CT/MRI) was performed significantly more frequently in patients subsequently diagnosed with malignant pathology than in those with non-malignant findings (7 (50.0%) vs. 10 (12.5%), p=0.003).

## Discussion

Pathology and malignancy rate

A meta-analysis of 17 studies published in 2016 reported an overall pooled cancer conversion rate of 8.8% (95% CI: 7.0%-10.7%) [8]. The proportion of urgent suspected head and neck cancer referrals diagnosed with malignancy in published studies ranges from 6% to 14.6% (Table 3).

**Table 3:** Malignancy detection rates from other ENT two-week wait (2WW) units in the literature

Authors	Sample size	Detection rate (%)
Rimmer et al., 2012 [9]	400	9
Douglas et al., 2019 [10]	2116	11.8
Williams et al., 2002 [11]	100	11
Lyons et al., 2004 [12]	171	14.6
Shah et al., 2006 [13]	150	6
East et al., 2005 [14]	48	6.3
Singh and Warnakulasuriya, 2006 [15]	76	7.9
Duvvi et al., 2006 [16]	187	10.2
Hobson et al., 2008 [17]	177	12.4
McKie et al., 2008 [18]	1079	10.9
Haikel et al., 2011 (audit 1) [19]	163	10.4
Haikel et al., 2011 (audit 2) [19]	542	9.8
Miller and Hierons, 2012 (audit 1) [20]	63	11.1
Miller and Hierons, 2012 (audit 2) [20]	49	6.1
Madhvani et al., 2012 [21]	252	7.9

The current study, on the other hand, detected cancer in 14.9% of patients through a combination of telephone consultation, use of a risk calculator, and radiological investigations - with figures comparable to those reported in the meta-analysis. Furthermore, pathology was identified in more than half of the patient cohort, as described above. Douglas et al. [10] and Rimmer et al. [9] reported benign pathologies in 26.8% and 40.3% of their cohorts, respectively.

Undoubtedly, clinical examination and direct visualisation remain vital components of head and neck assessment and cannot be entirely replaced by imaging. Nonetheless, modern radiological techniques are highly reliable in supporting diagnosis. For instance, ultrasonography has been shown to be more sensitive than clinical examination in detecting pathological cervical lymph nodes (96.8% vs. 73.3%) [22]. It also demonstrates a sensitivity of 95% and specificity of 83% in distinguishing metastatic from reactive lymph nodes [23]. Cross-sectional imaging, such as MRI, has a reported sensitivity of 98% for detecting primary head and neck tumours and 85% for identifying lymph node metastases [24].

Therefore, a combination of thorough history-taking, effective risk stratification, and selective radiological investigations - as employed in the TnT model - helps ensure that cancer diagnoses are not missed or delayed [25]. In cases where imaging is normal but symptoms remain concerning, patients can still be offered a face-to-face review. All discharged patients were given safety-netting advice, and general practitioners (GPs) were advised to re-refer if symptoms persisted or worsened.

Resource implication

Rimmer et al. and Douglas et al. referred 37.5% and 57% of their patients from 2WW referrals for radiological investigations following consultation, respectively. In contrast, only 17% (n=495) of patients in our study were referred for imaging using the TnT model [9,10]. Despite referring significantly fewer patients, our malignancy pick-up rate remained comparable to those reported in the literature.

Several factors may explain this difference, including population demographics, catchment area variation, timing of patient presentations during the pandemic, clinician discretion in ordering scans, and the adoption of a more stringent imaging referral approach during the COVID-19 period. However, direct comparisons between studies should be made with caution due to these inherent differences.

In addition to the malignancies detected, 40.40% (n=38) of the TnT cohort (n=94) were found to have benign pathology. This further validates the clinicians’ decisions to refer these patients for radiological investigations, also providing reassurance to both patients and referring clinicians.

It is acknowledged that nearly half of the patients (n=42, 44.70%) had normal imaging results, which contributed to a modest impact on radiology services. This is likely attributable to the absence of clinical examination and the heavy reliance on patient-reported symptoms or referral narratives provided by GPs. However, it remains unclear whether face-to-face consultations would have significantly reduced this proportion. Importantly, the potential strain on radiology services was counterbalanced by the reduced number of face-to-face clinic appointments and decreased hospital footfall during the pandemic.

With the stringent application of the HaNC risk calculator and sound clinical judgement, diagnostic accuracy could potentially be further improved in the future by incorporating additional tools, such as virtual or video consultations.

Waiting-times effectiveness

From a service delivery perspective, the initial telephone triage enabled 104 (21.0%) of the 495 patients referred through the urgent suspected head and neck cancer (2WW) pathway to be discharged following a single telephone consultation with appropriate safety-netting advice. Subsequently, among the 94 patients managed through the telephone-and-test pathway, 49 (52.1%) were discharged following radiological investigation without requiring further outpatient review. In comparison, Rimmer et al. and Douglas et al. reported discharge rates of 37.5% and 13.4%, respectively, after conventional face-to-face assessment and subsequent investigation [9,10].

Hence, the TnT model not only reduces the time to diagnosis and/or treatment but also decreases the number of hospital visits. This is particularly important in an ENT department with overwhelmed two-week wait cancer services, as it facilitates achievement of the Faster Diagnosis Standard (FDS) and allows more timely appointments for high-risk patients who would otherwise face longer waiting times.

Moreover, earlier discharges also shorten future outpatient waiting times. Other specialties, such as general surgery, have shown that using a straight-to-test approach via gastroscopy or colonoscopy reduces the number of outpatient appointments arising from 2WW referrals, thereby reducing waiting times for diagnosis and treatment [25].

Limitations of the study

While the TnT model has shown promising results, numerous limitations must be acknowledged to fully appreciate its context and guide future implementation. One of the key limitations of the model is the small sample size, which limits its statistical power and the absence of a conventional face-to-face comparison group that would strengthen its results and clinical implications. Moreover, the use of the HaNC-RC may be more specific for certain head and neck cancers but less so for thyroid malignancies, resulting in their inadvertent classification as low-risk. Reassuringly, none of the patients discharged following telephone consultation and imaging have, to date, re-presented with a subsequent diagnosis of malignancy.

Additionally, the lack of physical clinical examination at the stage when investigations are initiated creates its own challenges, such as the risk of selection bias and subsequently either over-investigation or under-triage, depending on the clinician's interpretation and the patient's ability to communicate. This might result in increased pressure and capacity issues in already stretched radiology services and could delay investigations for higher risk patients.

It is worth noting that, in our service, all consultations and triage decisions were carried out by consultants, whereas current telephone triage services are increasingly nurse-led. This shift may introduce a degree of inter-observer variability or selection bias, particularly in the early stages of implementation. However, with appropriate training, use of validated risk stratification tools such as the HaNC-RC, and accumulation of experience over time, this variation can be minimized, ensuring safe and consistent triaging across different staffing models.

Future perspectives

We believe this pilot model has the potential to be safely utilized in the hospital setting. After analyzing our results and reflecting on the lessons learned during COVID-19, we conclude that this model is the one we aim to integrate within our pathways. Given the advantages, we are now planning to implement the TnT approach within our newly introduced Telephone Assessment Clinic (TAC) appointments. By applying this model to a larger cohort in our current practice, we aim to generate high-volume data that will further validate its safety and effectiveness, ultimately supporting its wider adoption in routine practice.

## Conclusions

The telephone-and-test model appears to be a proactive and practical pathway in the current climate of rising referral volumes and increasing pressure to meet the FDS. As demonstrated in our pilot study, it provides a reliable approach for triaging urgent suspected head and neck cancer referrals and shows promise in improving triaging efficiency. The combination of structured telephone consultations, risk stratification using the HaNC-RC, and targeted radiological investigations facilitates the early identification of clinically significant pathology while maintaining a high malignancy detection rate.

Importantly, almost half of the patients referred directly for imaging were discharged following completion of their investigations, substantially reducing the need for unnecessary face-to-face consultations. This, in turn, decreases outpatient clinic demand, reduces hospital footfall, and enables more timely access for patients requiring urgent specialist assessment. Furthermore, the pathway appears to place only a modest additional demand on radiology services, while offering potential benefits in reducing waiting times and improving overall pathway efficiency.

Given the ongoing pressure on outpatient services and the continued increase in 2WW referrals, the TnT model appears to be a feasible and scalable strategy to consider for the optimisation of the head and neck cancer pathways. Larger prospective studies are warranted to validate these findings and assess the generalisability of this approach across different healthcare settings.
